# Heterogeneity as a feature: unraveling chromatin’s role in nuclear mechanics

**DOI:** 10.1080/19491034.2025.2545037

**Published:** 2025-08-21

**Authors:** Wessel S. Rodenburg, Amy R. Strom, Jorine M. Eeftens

**Affiliations:** a Institute for Molecules and Materials, Radboud University, Nijmegen, The Netherlands; b Radboud Institute for Molecular Life Sciences, Radboud University, Nijmegen, The Netherlands; c Department of Chemical and Biological Engineering, Princeton University, Princeton, NJ, USA

**Keywords:** Chromatin, genome organization, heterogeneity, multiscale quantification, nuclear mechanics

## Abstract

Mechanical forces are a ubiquitous feature of the cellular environment. These forces propagate to the nucleus, where the mechanical response is critical for cellular function and survival. In addition to the nuclear lamina and cytoskeletal connections, chromatin is a key structural and mechanoresponsive element which not only contributes to bulk stiffness but also dynamically adapts its organization in response to mechanical stress. Crucially, chromatin is not a uniform material – its organization and mechanical properties vary across time, cell state, and even within individual nuclei. This heterogeneity underpins compartmentalization, gene regulation, and potentially, disease states when disrupted. In this review, we summarize recent experimental advances that have illuminated chromatin’s role in nuclear mechanics, emphasizing the importance of heterogeneity. We argue that an integrated, multiscale, and quantitative framework is essential for dissecting chromatin’s mechanical contributions. By doing so, the field will be better positioned to link nuclear mechanics to functional biological outcomes.

## Introduction

**Figure 1.**
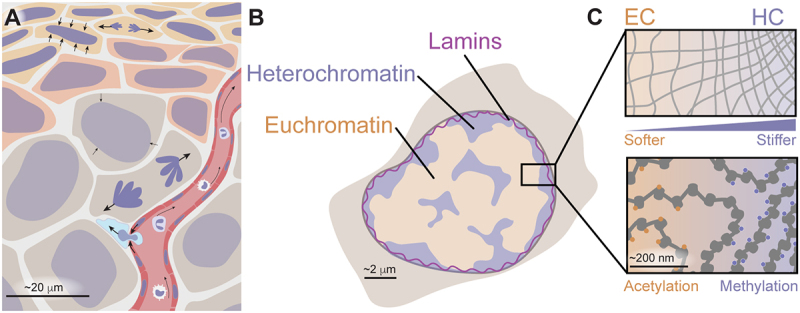
Multiscale contributions of chromatin to nuclear mechanics A. Within tissues, cells are subjected to a range of mechanical forces that are transmitted to the nucleus. These forces vary depending on cell type, cell cycle stage, and physiological or pathological state. B. The mechanical response of the nucleus arises from multiple components, including the nuclear lamina, heterochromatin, and euchromatin, each contributing differently depending on the magnitude and duration of the applied force. C. The nuclear interior can be thought of as a gel with regions of differential density and crosslinking (top), driven by nuclear factors including chromatin (bottom). Euchromatic (EC) regions are typically acetylated, transcriptionally active, and decondensed, contributing minimally to nuclear stiffness and viscoelasticity. In contrast, increased levels of H3K9me3 or H3K27me3 promote chromatin compaction, enhancing stiffness of heterochromatic (HC) regions. Together, the spatial and chemical organization of chromatin and the structural scaffold of lamins define the mechanical behavior of the nucleus across timescales and deformation regimes.

Mechanical stress is a constant in the cellular environment in vivo. Circulating blood and immune cells are subjected to shear forces, migrating cells encounter spatial confinement, and tissue-resident cells experience persistent compression (Figure 1A). These mechanical deformations can propagate to the nucleus, causing it to deform [1,2]. The nucleus plays a central role in sensing and responding to these physical cues, and its ability to adapt is critical for maintaining homeostasis [3–6].

While nuclear mechanics have traditionally been attributed to the lamina and connection to the cytoskeleton, in recent years, chromatin has emerged as a key structural and mechanoresponsive element [7–10]. Chromatin occupies up to 52% of the nuclear volume and is thus a major structural component, strongly contributing to the overall stiffness of nuclei and acting as a primary force-bearing element [11,12] (Figure 1B). Beyond its structural role, chromatin actively adapts its organization in response to mechanical cues across multiple scales. On the mesoscale, this affects chromatin compartmentalization. Forces at this scale arise from active processes including transcription and interaction with biomolecular condensates, resulting in repositioning of domains or modulation of the local viscoelasticity (Figure 1C). On the molecular scale, these changes in viscosity influence diffusion and activity of chromatin-interacting factors including translocating motors [13]. Thus, chromatin integrates mechanical signals into structural and functional adaptations across length scales [14].

Importantly, chromatin’s non-equilibrium nature strongly influences mechanics across scales. Chromatin composition and organization are dynamic, varying over time and with epigenetic state, for example, during the cell cycle as the genome replicates and compacts into mitotic chromosomes, or during embryonic development as pluripotent cells transition toward differentiation [15–17]. These processes introduce substantial heterogeneity in chromatin structure across a cell population, leading to corresponding variability in nuclear mechanical properties. At the same time, heterogeneity exists within individual nuclei: local stiffness, viscosity, and mechanical responsiveness vary widely depending on chromatin compaction, transcriptional activity, and molecular interactions (Figure 1C) [18,19]. This interior mechanical heterogeneity is thus tightly linked with functional compartmentalization. Loss of this mechanical heterogeneity could disrupt nuclear organization and gene regulation, potentially contributing to the development of disease. Chromatin-driven mechanical diversity is therefore present both between cells and within each nucleus, reflecting different roles in functional organization.

Understanding chromatin’s complex role in nuclear mechanics thus requires an approach that spans multiple time and length scales. By accounting for the inherently non-equilibrium nature of the nuclear environment and developing robust methods to quantify mechanical properties, the field can advance toward a more integrated view of chromatin’s role in mechanics. Such insights are critical to move toward understanding how chromatin mechanics contributes to diseases, including cancer and developmental disorders. Here, we review how the field has experimentally approached the role of chromatin in nuclear mechanics over multiple scales and provide perspectives for future opportunities.

## Chromatin in nuclear stiffness and deformability

**Figure 2.**
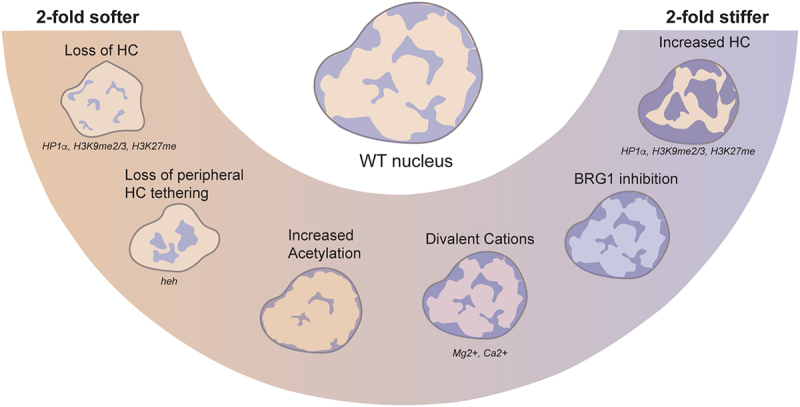
Effectors of chromatin mechanics. Loss of heterochromatin (HC)-associated factors—including HP1α, H3K9me2/3, H3K27me, loss of the INM-anchoring protein heh, and increased levels of histone acetylation results in an approximately 2-fold decrease in nuclear stiffness. Conversely, nuclear stiffness increases by up to ~2-fold upon treatment with divalent cations, inhibition of BRG1 (the catalytic subunit of the SWI/SNF chromatin remodeling complex), or increased levels of heterochromatin-associated marks such as H3K9me2/3. Together, these perturbations illustrate how chromatin compaction state, localization, and biochemical modifications modulate nuclear mechanics.

**Figure 3.**
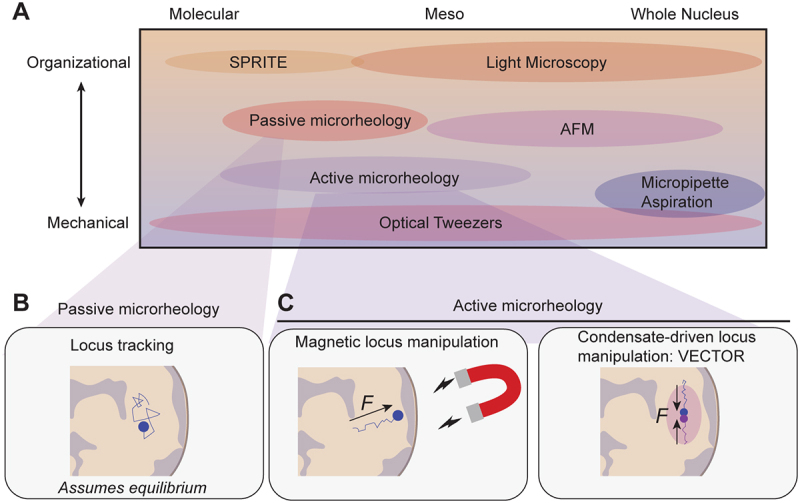
Quantitative techniques for nuclear mechanics. (A) Overview of methods used to probe nuclear organization and mechanics across scales. (B) Passive microrheology, such as locus tracking and diffusion-based models, assumes thermal equilibrium. (C) In contrast, active rheology techniques introduce controlled perturbations and enable modeling of nonequilibrium mechanical responses at the mesoscale.

Chromatin strongly contributes to the stiffness and deformability of nuclei [20]. It is thus intuitive that changes in the composition, organization and architecture of chromatin are reflected in the mechanical properties of the nucleus (Figure 2). Several methods have been employed to observe these changes [21], typically by applying an external force to the nucleus, either in isolation or in whole cells, and quantifying its deformation over time (Figure 3A). From the resulting force-deformation curves, the stiffness of the nucleus is extracted in the form of the spring constant (force over deformation) or Young’s modulus (stress over strain). Additionally, material properties can be derived through stress-relaxation or creep tests, or by applying oscillatory forces. The most widely used techniques are atomic force microscopy [22–24], optical and magnetic tweezers [25–27], and micropipette aspiration (Figure 3A) [28].

A general intuition is that densely packed chromatin regions are more resistant to deformation than loosely packed areas. Bulk chromatin compaction can be induced by exposing nuclei to divalent cations such as Ca^2+^ and Mg^2+^. Consistent with this idea, cation-induced compaction reliably stiffens nuclei in micropipette aspiration studies [16,29,30]. An alternative approach to affect chromatin compaction is through altering histone-tail modifications. Bulk enzymatic cleavage of histone tails leads to decompaction, and softens isolated nuclei as measured with optical tweezers [31], but more specifically, up- or downregulation of specific modifications on histone tails changes compaction state, thereby tuning nuclear mechanical properties. Acetylation of histone tails is generally associated with decondensed chromatin as typically found in euchromatin regions. Accordingly, nuclei become up to roughly two-fold softer as the extent of histone acetylation increases [7,8,29,32]. The opposite effect, stiffer nuclei upon increasing heterochromatin through histone methylation levels, is also observed [8]. The observed nuclear stiffening was attributed to an increase of constitutive heterochromatin marks H3K27me3 and H3K9me3, typically found in heterochromatic regions [8]. Evidence in support of this hypothesis is provided by specific inhibition of SUV39H1 which mediates H3K9 trimethylation, resulting in nuclear softening by nearly two-fold [5,33]. Similar effects were found in isolated yeast nuclei. Nuclei lacking H3K9me2/3 demethylase were stiffer, whereas nuclei lacking H3K9 methyltransferase were softer [34]. Together, these studies show that nuclear stiffness can be regulated by modulating the compaction state of chromatin (Figure 2).

Chromatin is further shaped and organized by chromatin-binding proteins that often interact with histones and their modifications. Several of these proteins have been shown to alter nuclear mechanics, but it is difficult to disentangle the contribution of these proteins themselves, versus their downstream effect on epigenetic state. One such protein is HP1α, a histone methylation reader that has also been implicated in the establishment and maintenance of histone methylation and heterochromatin [35,36]. As measured with micropipette aspiration, rapid depletion of HP1α reduced the mechanical strength of isolated nuclei by roughly two-fold [37]. It was tested if this effect was attributed to altered levels of H3K9-methylated histones, but importantly, this mechanical role was shown to be independent of histone methylation and heterochromatin formation. This indicates that HP1α has a structural role that impacts nuclear mechanics that is independent of chromatin compaction through histone modifications, as was also shown in vitro [38]. A similar key mechanical contribution was described for Swi6 (HP1 homologue in yeast) [34]. Interestingly, specifically the condensed, rather than the chromatin-bound Swi6 pool, was shown to impart nuclear stiffness [34]. These findings suggest a separate role for HP1-like proteins in maintaining nuclear stiffness, potentially providing mechanical support through condensate formation [38].

Nuclear mechanics may also be regulated by the localization of chromatin, specifically peripheral chromatin. A dense layer of heterochromatin is typically present directly adjacent to the nuclear periphery, which is physically tethered to the nuclear membrane through connections with inner nuclear membrane proteins and the nuclear lamina [39,40]. A study in yeast showed that tethering of chromatin to the periphery supports mechanics of the nucleus (Figure 2) [26]. Nuclei lacking the inner nuclear membrane (INM) chromatin-binding protein *heh2* softened by ~1.5-fold, and showed strongly reduced effective viscous drag coefficient from ~2 to 0.5 $\mathrm{pN}\cdot s\cdot nm^{-1}$[26]. Nuclei lacking other INM chromatin-binding proteins *heh1* and *ima1* showed a similar but milder effect on nuclear stiffness and viscosity [26]. However, as yeast lack lamins, it is unclear how this translates to mammalian nuclei, where lamins provide essential tethering points for chromatin. The interplay between chromatin and lamins makes it challenging to experimentally disentangle their individual contributions to nuclear mechanics, as chromatin perturbations may also indirectly influence interactions with the lamina [7]. Alternative chromatin organizational structures, such as the ‘inverted’ phenotype of rod photoreceptor cells [41], could be an exciting avenue to explore this connection.

## Chromatin adaptations in response to force

Chromatin not only serves as an important structural component of the nucleus but also acts as a mechanoresponsive scaffold, modulating its architecture [5] and/or gene expression programs [42] in response to mechanical cues. Forces may directly induce nuclear deformations [2], or may be indirectly propagated to the nucleus through the cytoskeleton [43] and/or other mechanosensitive components such as force-sensitive ion channels [5], each triggering chromatin adaptations.

Changes in chromatin compaction and epigenetic state have been found in response to substrate stretching [5,44–48], cell compression [49], and constricted migration [50–54]. However, the reported effects of these mechanical perturbations on chromatin are variable. Force has been found to both increase heterochromatin marks [49,52,53], decrease heterochromatin marks [5,54,55], switch from H3K9me2/3 to H3K27me3 [47], and spatially redistribute heterochromatin regions [48,51]. The discrepancies in these studies stress that chromatin’s response to force is highly variable. This is partially attributed to the method used to apply force [21], and the extent, duration, direction and nature (i.e. compression or stretching) of force application [21,56,57]. Quantitative techniques such as optical tweezers can potentially overcome this source of variability, as they can ensure that each cell or nucleus experiences the exact same force [58] (Figure 3). Excitingly, this opens up the possibility of using these precise manipulations to trigger predictable responses. Furthermore, large contributing factors to these different outcomes are the use of different cell types and inherent heterogeneity between cells. Next, steps toward understanding chromatin’s response to force should aim to understand this heterogeneous response as a functional feature, as it enables tissues and cells to adapt to diverse environments and fulfill specialized roles.

Physical cues also regulate chromatin indirectly through downstream signaling pathways. Local signals are sensed by integrins or stretch-activated receptors and transduced through secondary messengers, ultimately resulting in the activation of transcription factors that shuttle into the nucleus [59]. Several of such mechanosensitive transcription factors have been identified, including YAP/TAZ [60], MAL-SRF [61,62] and NF-κB [63]. It is currently not well understood if changes in chromatin composition reflect direct consequences of physical force, or of force-activated signaling pathways [56]. A number of hypotheses have been put forward. Physical force could lead to local DNA damage, which in turn recruits repair machinery and alters chromatin state [22,64,65]. Alternatively, force-induced deformations of the nucleus and associated endoplasmic reticulum can lead to an influx of calcium, which in turn can influence local compaction state and compartmentalization of chromatin modifiers [5]. Additionally, changes in actin dynamics upon mechanical stress may allow chromatin modifiers such as HDAC3 that are normally sequestered in the cytoplasm to accumulate into the nucleus [49,66]. Lastly, influx of mechanosensitive transcription factors might lead to altered nuclear properties by the presence of these proteins themselves, altered transcriptional activity, changes in histone marks, or local decompaction. Microscopy approaches that capture spatial and temporal changes in chromatin organization might help infer how chromatin responds to mechanical forces, potentially shedding light on underlying sensing mechanisms [56].

## Mesoscale chromatin mechanics

The current view of interior chromatin organization is that dynamic loops form within compartments of similar transcriptional activity [67]. Euchromatin compartments are open and transcriptionally active, whereas heterochromatin is compacted and transcriptionally silenced [68,69]. Their spatial location correlates with transcriptional status: heterochromatic domains often reside at the nuclear periphery or cluster around the nucleolus [40,70,71]. Indeed, this containment to distinct locally defined regions within the nucleus has been confirmed by genomic methods [72] (Figure 3A).

Reconciling this entrenched, compartmentalized structure of chromatin with its dynamic, mechanoresponsive nature remains a challenge [73]. Biomolecular condensates offer a compelling bridge, as their biochemical composition and physical properties are highly tunable and responsive to cellular cues. Condensates are ubiquitous within the nucleus, and their interactions with chromatin are essential to both their formation and function [74,75]. For instance, transcription initiation condensates are thought to promote gene expression, primarily associating with euchromatin [76,77], while repressive condensates localize to heterochromatin, where they may contribute to transcriptional silencing [78,79]. The preferential localization of condensates to specific chromatin compartments likely contributes to the establishment of selective inclusion and exclusion mechanisms, which can generate local forces. In addition, tethering of chromatin loci to condensates can modulate chromatin organization [80]. The material state of the condensate, ranging from liquid-like to gel-like, will subsequently affect the mechanical changes [81]. In this way, condensates may act as dynamic mediators that both sense and reshape the local chromatin environment, integrating structural stability with mechanical and functional adaptability.

A recent study by Strom et al. explores how condensate-driven forces can influence chromatin mechanics and position [82] by using engineered biomolecular condensates that form near specific chromatin regions. The fusion and dissolution dynamics of these condensates generates sub-pN interfacial forces on the chromatin, thereby repositioning nearby chromatin loci. A different method by Keizer et al. also succeeded in physically pulling loci through the nuclear environment [83]. Using magnetic nanoparticles targeted to a specific genomic locus, the locus was pulled in response to controlled magnetic pulse. Importantly, the chromatin trajectories exhibit scale-free behavior, indicating there is no single characteristic time- or lengthscale governing these dynamics. This suggests that chromatin behaves as a complex network, and forces over a wide range of scales can tune its mechanical response. Both methods showed relatively low forces (~1 pN) were sufficient to pull chromatin across variable chromatin densities, suggesting that loci are not rigidly fixed in space, even within structurally defined domains. The development of these techniques toward specific loci and applications will provide new insights into how chromatin responds to force [84].

These recent methods can potentially tackle another challenge: measuring inner nuclear viscosity, which so far has been challenging. A traditionally employed approach is passive microrheology [85] (Figure 3B). With this technique, the mean-squared displacement of a probe over time is used to deduce the diffusion coefficient, leading to viscosity calculation through the generalized Stokes-Einstein relation, which assumes thermodynamic equilibrium [86]. Several studies have used tracking techniques for single chromatin loci to monitor their mean-squared displacement [87–89]. These works concur that chromatin loci are subdiffusive, suggesting confinement. However, more meaningful measurements in active materials such as living cells require driving the system out of equilibrium. This could be achieved with active microrheology, where the probe is forced through the material, setting fluid and particles in motion (Figure 3C). It is notoriously difficult to perform active rheology in living cells, primarily because the probes used to date are too invasive, with probe injection leading to low survival rates and impacting mechanical fidelity [90–93]. The minimally invasive probes in the form of magnetic nanoparticles or condensates used by Keizer and Strom circumvent this issue [82,83] (Figure 3C). By monitoring the displacement of loci over time as described, nuclear viscosity can be inferred or estimated, distinguishing between elastic (chromatin-network) and viscous (nucleoplasmic) contributions. Both methods measure viscosity in a similar range (~10^3^-10^4^ pa⋅s). However, it is again emphasized that mechanical properties are not uniform, even within a single nucleus. This intranuclear heterogeneity has functional implications, regulating how different regions react to mechanical force [23]. A combination of these new methods with advanced imaging techniques (Brillouin microscopy, elastography, FLIM, etc.) will enable visualization of the intranuclear heterogeneity [19,94], working toward an understanding of how processes such as transcriptional activity and local nucleosome stacking direct these properties, as this is currently limited.

## Connecting molecular actions to bigger consequences

Interactions between nucleosomes play a critical role in chromatin compaction and organization. *In vitro* force-spectroscopy studies using reconstituted nucleosomal arrays have robustly quantified the forces at which nucleosome stacking and wrapping are disrupted to be 5-10 pN [95–99]. The same reconstituted chromatin fibers, typically consisting of 12 nucleosomes, have been shown to form condensates in a variety of conditions [100,101]. Both unstacking forces and propensity to form condensates depend on the presence of linker histones, nucleosome repeat length, histone modification, ionic concentrations, and protein interactions. The emerging hypothesis is that formation of tightly stacked chromatin increases the force required to disrupt this organization within the fiber, but prevents interactions between different fibers, resulting in reduced propensity to form higher-order condensates. The parallels in experimental set-up between these two length scales allow for interesting multi-scale interpretations, including the range of various factors that influence this organization, hinting at what could be the source of variable displacement forces in the heterogeneous nuclear interior. It should, however, be considered that the majority of these in vitro characterizations on both the molecular and condensate scales are performed on homogeneous nucleosomal arrays. All octamers are assembled on repetitive sequences with equal spacing, which does not reflect the situation in live cells. Incorporating this molecular heterogeneity is a logical next experimental step.

The last decade, the single-molecule field has made great progress in understanding the molecular mechanism of DNA-organizing motors. Structural Maintenance of Chromatin (SMC) proteins organize chromatin by extruding loops, fueled by ATP [102]. The formed loops could function as a way to bring regulatory elements such as promoters and enhancers into physical contact, thereby directly impacting gene expression, but more recent evidence suggests these loops are transient in nature [103]. In addition, loops and gene expression levels are largely maintained after depletion of SMC protein cohesin [104]. This again invites an alternative view that bridges the dynamic nature of chromatin with the structural organization into loops.

An interesting perspective is that the dynamic formation and disruption of these loops functions to maintain chromatin fluidity [105]. Compartments collapsed into phase-separated compartments are difficult to penetrate for certain factors, and spontaneous escape of chromatin loci is unlikely as it requires a few pN. ATP driven SMC motors could help fluidize this organization. SMC proteins have a sub-pN stalling force, meaning that they cannot extrude loops against forces higher than 1 pN [106–108]. This is close to the force required for actively pulling a locus across the nucleus, indicating that loop extrusion and locus pulling could aid one another to reorganize chromatin. This idea of SMC motors as compartment disruptors furthermore implies that their molecular action affects the fluidity and deformability of the whole nucleus. This connection is not entirely unlikely, as a similar link between motor activity and fluidization exists for the SWI/SNF/BAF chromatin-remodeling complex. Early single-molecule experiments demonstrated that this ATP-driven complex can slide along DNA and reposition or evict nucleosomes [109–111], but recent work showed that when this activity is abolished in cells through chemical inhibition of the BRG-1 motor subunit, nuclei stiffened [112] (Figure 2). Furthermore, these nuclei showed decreased dissipation when subjected to force, indicating a loss of nuclear fluidity in the absence of this active remodeling process [112]. Which and how active processes contribute to chromatin fluidization is testable, so future work will refine and revise this model, connecting the molecular scale back to the nuclear scale.

## Conclusion and perspective

To further understand the role of chromatin as force responder, a key ambition should be to shift to research grounded in quantitative measurements across multiple spatial and temporal scales, with a critical emphasis on heterogeneity – both between different nuclei and within the nuclear interior. Chromatin is not a passive scaffold, but a dynamic organelle that shapes the physical properties of the nucleus. These mechanical properties influence and respond to key nuclear processes, but they cannot be understood without measuring the forces, displacements, and material responses involved. These properties are not uniform across cell-types and have considerable spatial and temporal variability. We must not treat this heterogeneity as noise, but as a fundamental feature. Only multi-scale quantitative approaches can capture this complexity and reveal how chromatin mechanics serve to function.

## Data Availability

Data sharing is not applicable to this article as no new data were created or analyzed in this study.
